# Case report: surgical removal of a migrated needle in right ventricle of an intravenous drug user

**DOI:** 10.1186/s13011-017-0134-1

**Published:** 2017-12-08

**Authors:** Xianming Fu, Kai Chen, Xiaobo Liao, Kangjun Shen

**Affiliations:** 1 Department of Cardiovascular Surgery, The Second Xiangya Hospital, Central South University, Changsha, Hunan People’s Republic of China; 2 Mental Health Institute of The Second Xiangya Hospital, Central South University, Changsha, Hunan People’s Republic of China; 3 Department of Cardiovascular Surgery, The Second Xiangya Hospital, Central South University, Changsha, Hunan People’s Republic of China; 4 Department of Cardiovascular Surgery, The Second Xiangya Hospital of Central South University, 139. Renmin Road, Changsha, Hunan 410011 People’s Republic of China

**Keywords:** Surgery, Migrated needle, Illicit drug abuse, Drug rehabilitation treatment

## Abstract

**Background:**

Illicit drug use has become an increasing public health and social concern in the past decades worldwide. Intravenous injection has an elevated risk of infection. Needle embolism is a rare complication of intravenous drug users, Retained broken needles can lead to local complications, such as infection, but they also have the potential to embolize to heart or lung, and lead to serious complications.

**Method:**

We reported a rare case of an intravenous drug user which a retained broken needle fragments in the inferior wall of the right ventricle.

**Results:**

We performed a successful surgery and give our comments and recommendations for illicit drug use worldwide and in China.

**Conclusions:**

Illicit Drug use becomes a global problem because of its health and social harmfulness. To help drug addicts and provide prevention and treatment services are the obligations and responsibilities of all medical workers.

## Background

Illicit drug use has attracted more social concern and become a public health concern in the past decades worldwide. Intravenous drug users has an higher risk of infection. Needle embolism, as a rare complication of intravenous drug users [1], can lead to local complications, such as infection. Retained broken needles have the potential to become the emboli in heart or lung, and lead to serious complications. Here we reported a rare case which a retained broken needle fragment in the inferior wall of the right ventricle, we performed a successful surgery and give our comments and recommendations for illicit drug use in China.

## Methods and results

**Fig. 1:**
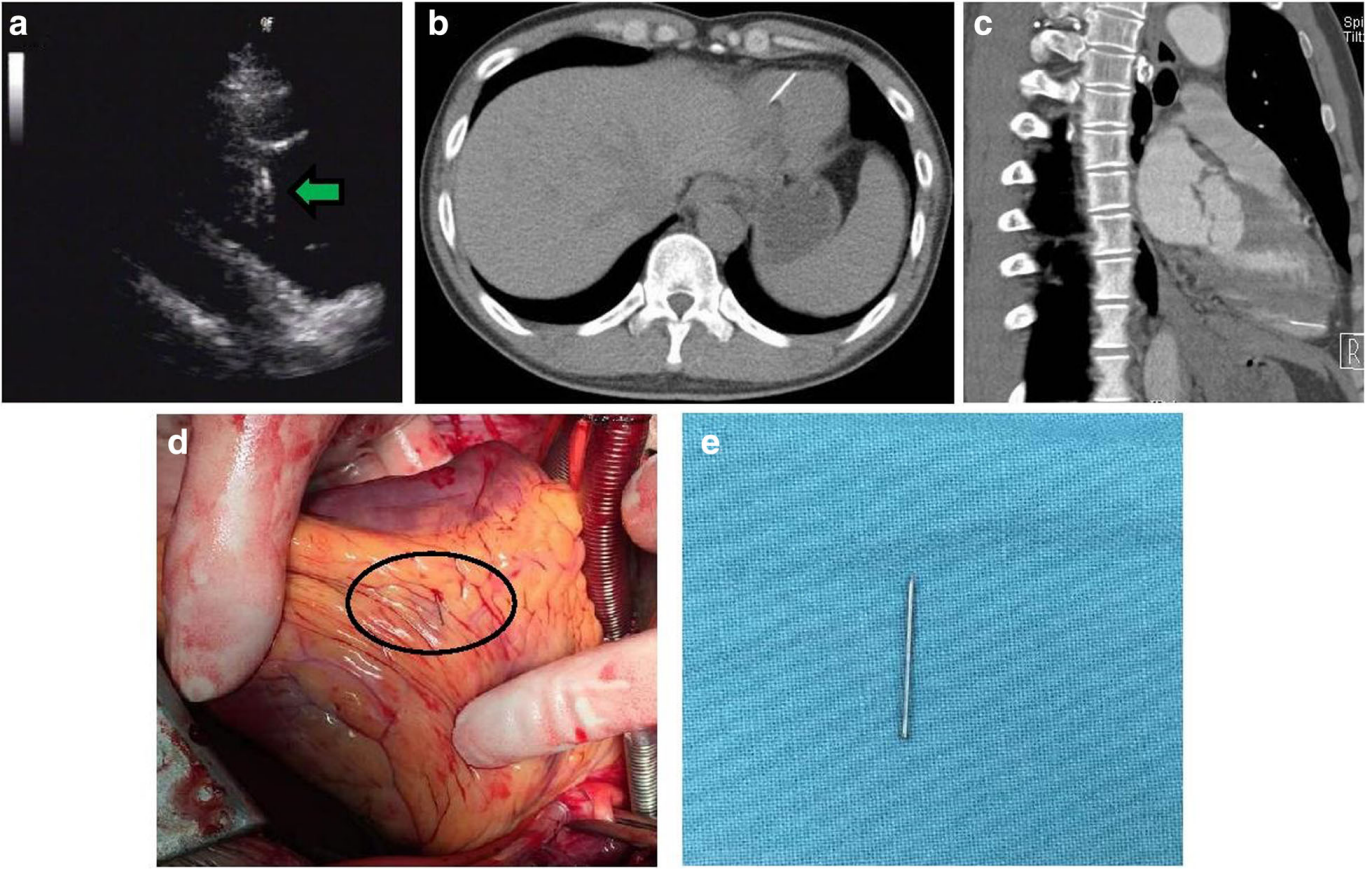
Retained needle fragment in the right ventricle. **a** Transthoracic echocardiography revealed a retained needle fragment in the inferior wall of the right ventricle, and no detectable pericardial effusion. **b** and **c** Computed tomography confirmed the location of a needle in the inferior wall of the right ventricle and near the ventricular septum. **d** and **e** The needle was found and removed from the right ventricle

A 40-year-old man presented to the emergency department after a needle broke in his right groin while taking heroin intravenously 20 days ago. There were neither special symptoms nor evidence of infection or infarction. Vital signs and electrocardiogram were normal, and he was hepatitis C virus (HCV) positive, a heavy user of heroin and cocaine over 20 years. Transthoracic echocardiography revealed a retained needle fragment in the inferior wall of the right ventricle (Fig. 1a), and no detectable pericardial effusion. Computed tomography confirmed the location of a needle in the inferior wall of the right ventricle and near the ventricular septum (Fig. 1b and c). Emergency open-heart surgery through a median sternotomy under cardiopulmonary bypass was performed. Chest roentgenogram was performed by the moving bedside X-ray machine in the operation room to rule out unsuspected migration of the needle during patient positioning before proceeding with an incision. The needle was found and removed from the inferior wall of the right ventricle (Fig. 1d and e).The patient recovered uneventfully after the surgery and was discharged home on postoperative day 6.

## Discussion

Needle embolism is an infrequent complication of intravenous drug users (IDUs) [1]. Broken needles occur most commonly when the needle separates and comes apart from the hub. Inflammation and infection after needle embolism are relatively common in local wound site. While the central embolim of needle fragmentscan potentially cause serious complications such as cardiac perforation, pericarditis, infective endocarditis, arrhythmias, and pulmonary abscess [1]. In most cases, it is recommended to remove the needle once the embolism is found in order to prevent delayed complications [2].

Illicit drug use is an intractable and global public health problem. According to the *World Drug Report 2016* [3], 250 million of people between the ages of 15 and 64 years used drugs in 2014. Over 29 million people who used drugs suffer from drug use disorders, of whom 1 in 6 is in treatment. What’s worse, the most current data available showed that 12 million people had a history of injecting drugs globally [4].Compared with the traditional way of taking drugs, IDUs are exposed to more health-related harms and have a high risk of premature death. Poor health conditions, unsterilized injecting sites and drug dissolving agents are the leading causes of bacterial infection. Because of poverty or convenience, sharing and repetitive use of needles is a common phenomenon which is the main cause of Human Immunodeficiency Virus (HIV) and HCV infection. About half of IDUs are estimated to share needles worldwide [5]. A recent surveillance surveys among IDUs in China also showed that 47.7% reported ever sharing needles [6]. The statistics from World Drug Report 2016 shows that 14% of IDUs are living with HIV and 50% with hepatitis C in 2014 [3].Several studies have shown that IDUs have a disproportionate prevalence (60–80%) of HCV infection globally [7–9]. The most current data available in China also showed that in 2011, 67% of IDUs are living with HCV [8]. In addition, studies have reported that people often have risky sexual behaviors after injecting opiates, resulting in the risk of HIV infection [7–9].

A cross-sectional survey study in United Kingdom published in 2002 found that 20% of IDUs had experienced a broken needle during the course of their injecting careers [10]. Given the great number of IDUs in China, subsequent needle embolization may be more common than it appears, particularly as drug misusers tend to avoid hospital unless seriously ill. Broken needles are hidden risks for IDUs and central needle embolism could even result in severe complications in heart and lung. Hence it is necessary to increase the familiarity with needle embolism, as an unusual but life-threating complication of IDUs.

In Mainland China and other districts in Asia, compulsory drug rehabilitation is a primary strategy for drug users. However, harm reduction approaches such as methadone maintenance treatment (MMT) and needle-syringe exchange projects (NSPs) are widely promoted in recent years. In Mainland China, a total of 767 methadone maintenance treatment clinics had been set up in 28 provinces by the end of 2014 [11].While in Changsha, the city where our hospital located, 1990 patients received treatment in a total of 3 MMT clinics in 2013 [12]. Meanwhile, needle-syringe exchange projects were pushed forward in Mainland China. In 2014, NSPs were held in 14 provinces, with 814 needle exchange sites. Over 56,000 drug users participated in the projects and more than 11 million needles and syringes were handed out [11].

It was necessary for this patient to have drug rehabilitation treatment after being discharged. We recommended him to attend MMT and NSP. According to several studies in China, MMT can significantly reduce criminal activity and improve employment rate and social well-being, that helps drug users to resume societal and familial functions [6, 13, 14]. And NSPs decreased levels of injecting frequency, repetitive use and sharing of injecting equipment among Chinese IDUs.

## Conclusions

Illicit drug use becomes a global problem because of its health and social harmfulness. To help drug addicts and provide prevention and treatment services are the obligations and responsibilities of all medical workers.

## Abbreviations

HCV: Hepatitis C virus
HIV: Human Immunodeficiency Virus
IDUs: Intravenous drug users
MMT: Methadone maintenance treatment
NSPs: Needle-syringe exchange projects
